# The Stress of Being Left Out: Physiological and Behavioral Responses to Ostracism in Preverbal Infants

**DOI:** 10.1111/infa.70128

**Published:** 2026-09-23

**Authors:** Giada Basset, Ermanno Quadrelli, Chiara Turati, Hermann Bulf

**Affiliations:** ^1^ Department of Psychology University of Milano‐Bicocca Milan Italy; ^2^ NeuroMI Milan Center for Neuroscience Milan Italy

**Keywords:** behavior, heart rate variability, infants, ostracism, physiological reactivity

## Abstract

Being ostracized is a stressful experience that affects behavioral and physiological reactivity in adults. However, evidence regarding its impact early in life remains limited. This study investigated whether inclusion or exclusion during a ball‐tossing game influenced physiological and behavioral reactivity in 8‐month‐old infants (*N* = 59; 24 girls and 35 boys). It also explored whether parent‐infant interaction dynamics and parental mind‐attuned comments modulate infants' physiological responses, fostering greater adaptation to the social context. Results highlighted that excluded infants showed reduced heart rate variability (indexed via the Root Mean Square of Successive Differences, RMSSD), reflecting a heightened stress response, compared to included infants. They also displayed lower positive emotionality, higher negative emotionality, and greater active engagement, suggesting that sensitivity to ostracism emerges early in life. Exploratory analyses also revealed a modulatory role of parental behavior in shaping infants' physiological responses. Overall, these findings highlight that, since early life, infants exhibit physiological and behavioral reactions to ostracism, in line with the hypotheses that a system to detect and respond to social exclusion is present since the early stages of development.

## 1 Introduction

Early experiences play a crucial role in shaping the infant's brain, behavior, and psychological functions, with long lasting effects across multiple aspects of development (Cicchetti 2010; Pollak 2005). To effectively adapt to a dynamic environment and cope with stressful events, human beings rely on a flexible set of psychophysiological and behavioral responses (Bazhenova et al. 2001), that gradually emerge during development (Conradt and Ablow 2010).

The term stress denotes the psychological response that arises when an individual perceives a situation as threatening or challenging. This response is often adaptive, eliciting a cascade of behavioral and autonomic adjustments aimed at coping with the perceived challenge (K. E. Smith and Pollak 2020). A critical component of this response involves the autonomic nervous system, particularly parasympathetic activity, which is typically indexed by heart rate variability (HRV) as a proxy for vagal tone (Berntson et al. 1997). According to Porges' Polyvagal Theory (Porges 2001), vagal tone serves as a physiological marker of the organism's ability to regulate internal states and adapt to environmental demands. Changes in vagal tone from baseline to stress‐inducing conditions are thought to reflect the organism's regulatory flexibility, with greater vagal withdrawal (i.e., suppression) indicating heightened stress reactivity (Porges 1995).

Since early stressful experiences can have long‐lasting effects on development (K. E. Smith and Pollak 2020), the past decade has seen growing developmental research examining infants' behavioral and physiological reactivity to stressful situations, including the disruption of social interactions (Feldman et al. 2010; Haley and Stansbury 2003). A widely used paradigm in this field is the Still‐Face procedure, in which a mother initially engages in free interaction with her infant, then temporarily suppresses social communication by maintaining a neutral, unresponsive still‐face, and subsequently resumes normal play (Feldman et al. 2010). For example, a study from Bazhenova et al. (2001) has shown that at 5 months of age the Respiratory Sinus Arrhythmia (RSA), a widely used index of HRV, decreases in response to the transition from a free play condition to the Still‐Face episode. Research indicates that infants respond to a situational stressor, such as maternal unresponsiveness, with both physiological and behavioral changes, including reductions in vagal tone (e.g., HRV), altered cortisol levels, and increased negative affect (Bazhenova et al. 2001; Feldman et al. 2010; Haley and Stansbury 2003; Moore and Calkins 2004). Therefore, temporarily suppressing maternal availability, and disrupting social interaction, appears to elicit and alter both behavioral and physiological responses (Provenzi et al. 2016). On the other hand, literature has also shown that caregiver‐infant interactions play a crucial role in shaping infants' responses to stressful experiences. In particular, research has been focusing on Mind‐Mindedness, the tendency to interact and consider infants as individuals with a mind and to interpret their behavior in terms of underlying mental states (Meins and Fernyhough 2015). Findings indicate that early maternal comments that are non‐attuned (i.e., mismatched or poorly aligned with the infant's focus or affect) are associated with poorer recovery during reunion episodes following the Still‐Face paradigm (McMahon and Newey 2018). During reunion episodes, infants' biobehavioral reactivity and regulation, including heart rate and RSA, are also predicted by maternal capacity to accurately respond to the child's cues (i.e., maternal sensitivity; Conradt and Ablow 2010). Moreover, other studies have shown that higher levels of infant‐caregiver behavioral synchrony are associated with lower infant RSA at baseline and across all Still‐Face episodes (Busuito et al. 2019), further supporting the role of early interactive processes in infants' stress regulation.

Other lines of research have examined infants' behavioral reactivity to social stressors within more complex social settings, including triadic interaction contexts. For example, studies using a face‐to‐face ball‐tossing game have shown that not only dyadic disruptions, such as those in the Still‐Face procedure, but also triadic social challenges can elicit heightened negative emotional responses. Indeed, 13‐month‐old infants showed decreased positive emotionality and increased negative emotionality when ostracized (i.e., ignored and excluded) from a live triadic ball‐tossing game (Quadrelli et al. 2023, 2025) inspired by Williams and colleagues' Cyberball (2000), which is an online paradigm designed to manipulate social exclusion, where the ball is exchanged between the participant and two or more avatars controlled by the experimenter.

Previous studies with adults and older children have shown that humans have a fundamental need to belong, forming social connections across diverse contexts and generally resisting the dissolution of established relationships (Baumeister and Leary 1995). Within this framework, experiences of ostracism directly threaten fundamental social needs, including the need to belong, self‐esteem, perceived control, and meaningful existence. Such threats often prompt individuals to engage in behaviors aimed at restoring these compromised needs (Williams 2007) and are accompanied by considerable emotional distress (Williams and Nida 2022). Consequently, ostracism evokes a range of behavioral responses observable across development, from infancy to adulthood, including preschool and school‐age children (Basset et al. 2025; Mulder et al. 2023; Quadrelli et al. 2023; Testa et al. 2025; Williams and Sommer 1997), as well as cognitive changes related to social information processing and performance (Basset et al. 2025; Hawes et al. 2012; Kawamoto et al. 2014; Quadrelli et al. 2025).

Studies with adult participants have documented physiological stress responses to ostracism (Eres et al. 2021; Iffland et al. 2014; Kulakova et al. 2024). For example, Iffland et al. (2014) reported that adults exhibited an increased heart rate when ostracized during the Cyberball (i.e., after few initial equally distributed ball‐tossings, they were ignored and excluded by the avatars), and decreased heart rate when included. Similarly, it was found that excluded adult participants exhibited heightened cardiovascular and affective responses to a subsequent socially evaluative stressor (Williamson et al. 2018). Eres et al. (2021) observed that being excluded induced cardiovascular reactivity, specifically the diastolic and systolic blood pressure of adult participants increased during the Cyberball game. Finally, another study showed that during Cyberball the parasympathetic reactivity of healthy adult participants increased in the ostracism condition compared to the overinclusion condition (Kulakova et al. 2024). Collectively, these findings indicate that ostracism, even when simulated through an online ball‐tossing game, affects adults' cardiovascular and autonomic responses, potentially compromising stress regulation and emotional well‐being. Other studies have investigated this phenomenon in adolescents and children. For example, White et al. (2021) found that social exclusion in school‐age children was associated with event‐related cardiac slowing, a physiological marker linked to unexpected negative feedback. In contrast, another study reported blunted salivary cortisol response but no significant effects on heart rate or sympathetic nervous system activity in children and adolescents with obesity (Felnhofer et al. 2025). Additionally, it was shown that individual differences in resting‐state brain connectivity were related to more negative facial expressions during peer rejection, suggesting that baseline neural functioning may influence children's observable emotional reactions to ostracism (Mulder et al. 2025). However, research examining the physiological correlates of ostracism in children and adolescents remains limited. To date and to the best of our knowledge, no studies have investigated whether ostracism versus inclusion can induce psychophysiological stress during the first year of life, despite evidence that infants' physiological systems are shaped by the quality of social interactions (Bazhenova et al. 2001) and that they are capable of detecting and responding to disruptions in social exchanges, both in dyadic (Provenzi et al. 2016) and triadic (Quadrelli et al. 2023) contexts.

Considering the aforementioned premises concerning the impact of stressful experiences on infants' development and the documented effects of ostracism on physiological responsiveness, the primary aim of our study was to examine changes in infants' HRV to assess whether social exclusion elicits psychophysiological stress in preverbal, 8‐month‐old infants. As greater vagal withdrawal is recognized to be an indicator of heightened stress reactivity (Porges 1995), in our study we expect a significant decrease in HRV in the exclusion condition only, as this may reflect physiological distress (Hypothesis 1).

Moreover, by coding infant behavioral reactivity, we aim to assess how infants at 8 months of age react to a brief episode of social exclusion. Based on findings by Quadrelli et al. (2023, 2025), we expect excluded 8‐month‐old infants to show heightened negative emotionality, active engagement in the game and less positive emotionality. These behavioral patterns are anticipated to reflect their internal states and may indicate the disruption of basic psychological needs (Hypothesis 2). Importantly, it is known that 3‐month‐old infants can already engage in triangular attention with two social partners (McHale et al. 2008), and that earlier than 8 months infants exhibit greater negative emotionality when social interaction is disrupted (e.g., Bazhenova et al. 2001; Feldman et al. 2010). In addition, from 6 months of age infants start to sit on their own (Kretch et al. 2014; Martorell et al. 2006), being able to reach and grasp objects more easily, thus being engaged in visuomotor and playful activities (Hopkins and Rönnqvist 2002). By 8 and 9 months of age infants can judge whether an approaching ball can be caught, start moving their arms in time to intercept it (van Hof et al. 2008) and can anticipate others' intentional actions (Natale et al. 2014; Quadrelli et al. 2019). Taken together, these findings suggest that 8‐month‐old infants likely possess the cognitive and social skills to engage in a triadic ball‐tossing game and to detect exclusion from a social interaction. However, while we hypothesize that the behavioral findings reported by Quadrelli et al. (2023, 2025) in 13‐month‐old infants might be replicated, it is also possible that 8‐month‐olds will show different behavioral patterns, due to developmental differences between the two age groups, particularly in attention‐following abilities (Tang et al. 2024).

Our last goal was to explore whether parental Mind‐Mindedness, sensitivity and synchrony could affect infants' physiological activation throughout the experimental session. To this end, infants participated in a free‐play task with their caregiver prior to the ball‐tossing game, and we conducted exploratory analyses. Based on prior literature, we expected that parental mind‐attuned comments, responsiveness, and dyadic synchrony might be associated with infants' physiological regulation, as indexed by the Root Mean Square of Successive Differences (RMSSD), both in the free‐play and in the ball‐tossing game (Hypothesis 3). Specifically, infants whose parents express more mind‐attuned comments and are more sensitive might display better regulation. Indeed, it is known that infants' self‐regulation skills are deeply shaped by the quality of parent‐child relationship early in development (Taipale 2016). For instance, appropriate mind‐related comments have been linked to infants' physiological regulation during the first year of life (Zeegers et al. 2018), whereas sensitivity and synchrony are associated to infant vagal tone and emotional regulation (Puglisi et al. 2023; Rattaz et al. 2023). However, it could also be observed that infants with more synchronous caregivers tend to display lower RMSSD, as previous studies have shown that infants exhibit reduced parasympathetic activity in the context of higher behavioral synchrony. This pattern may reflect that infants with reduced parasympathetic activity require more coordinated engagement with their caregivers to support physiological and emotional regulation (Busuito et al. 2019).

## 2 Methods

**FIGURE 1:**
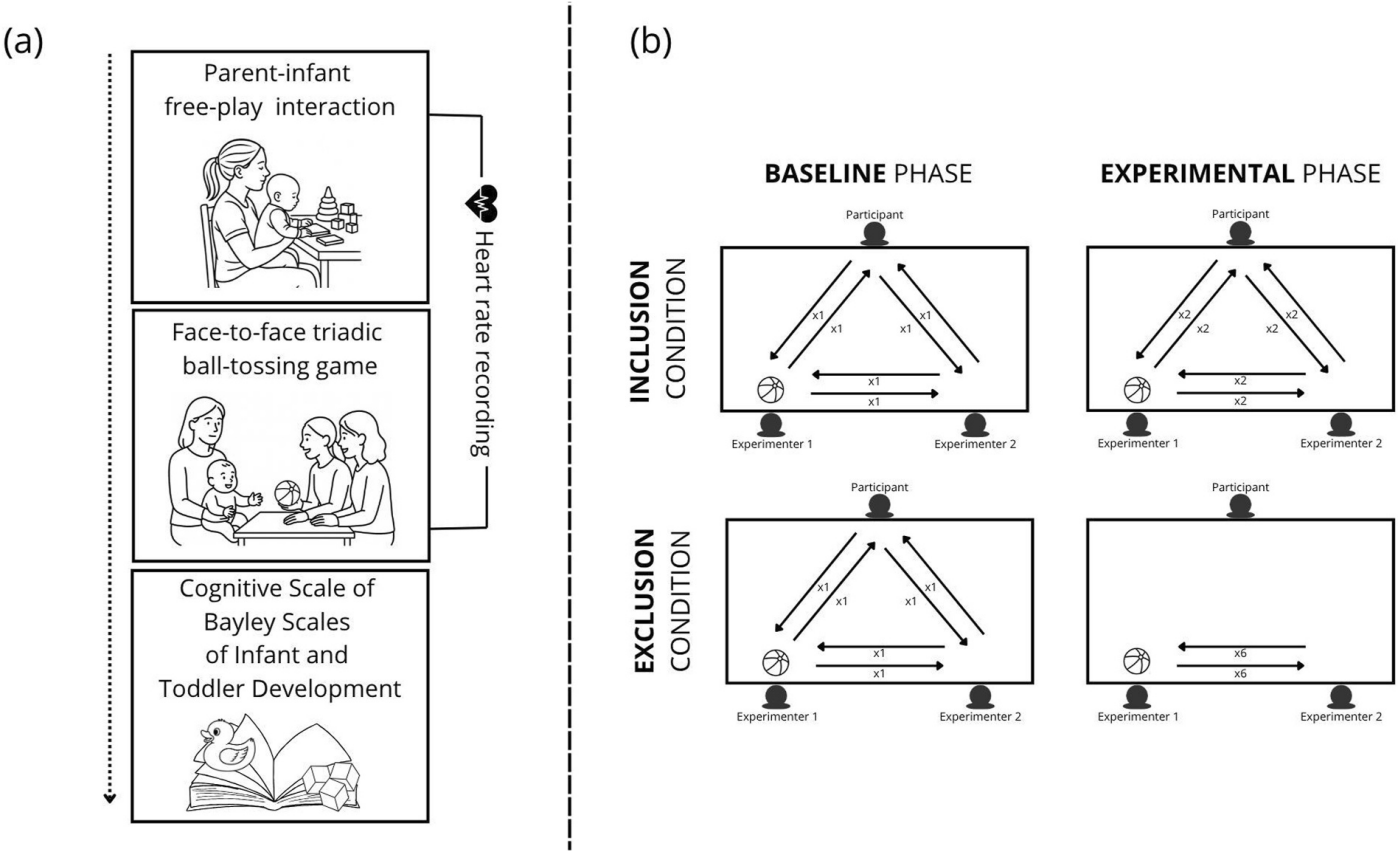
(a) Representation of the study procedure. Infants' heart rate was continuously recorded via ECG during both a free‐play session with their caregiver and a subsequent triadic ball‐tossing game where infants were either included or excluded. Both tasks were video‐recorded, and all infants completed the Cognitive Scale of the Bayley Scales of Infant and Toddler Development‐Third Edition. (b) Schematic representation of the triadic ball‐tossing game used in the current study. In the baseline phase, all participants equitably take part in the ball‐tossing game, both in the inclusion and exclusion condition. During the experimental phase the two conditions diverge. In the inclusion condition, participants continue to play equitably with the two experimenters, while in the exclusion condition, the two experimenters begin tossing the ball only between each other, ignoring the participant.

During the preparation of this work, the authors used ChatGPT (*Open AI*) to improve the writing style of the manuscript and to create the human‐like image shown in Figure 1a. The authors did not use AI for scientific content creation. After using this tool, the authors reviewed and edited the content as needed and took full responsibility for the content of the publication.

### 2.1 Participants

A total of sixty‐six 8‐months‐old participants were recruited and tested. To be accounted in the final sample infants had to meet these inclusion criteria: born full‐term (37–42 weeks of gestation), normal birth weight (> 2500 g) and no pre‐ or postnatal complications, as reported by the caregivers. Seven participants were tested but excluded from the final statistical analyses because they did not meet the inclusion criteria listed above (*N* = 6 in the inclusion condition) or due to fussiness (*N* = 1 in the exclusion condition). The final sample consisted of 59 participants (24 girls and 35 boys, *M*_age_ = 254 days, SD_age_ = 9.01 days, range = 235–271 days) with their caregivers (51 mums and 8 dads, *M*_age_ = 35.9 years, SD_age_ = 4.42 years, range = 30–50 years, mostly White). Infants were pseudo‐randomly assigned to either the inclusion (*N* = 30) or the exclusion condition (*N* = 29) by alternating their allocation to the experimental conditions prior to their arrival at the laboratory. This approach was adopted to minimize potential experimenter bias in the allocation of infants to conditions, while ensuring a balanced numerical distribution across groups.

Families were recruited from the metropolitan and suburban areas of Milan, encompassing a range of socioeconomic backgrounds. The primary language spoken at home was Italian. Information on the caregiver's background, infants' birth order and the presence of a second language spoken at home was collected (Table 1). Systematic information about infants attending daycare was not collected. However, considering the geographical area where the research was conducted (i.e., Northern Italy), childcare services are available from as early as 3 months of age, and national data indicate increasing enrollment rates and a gradual shift toward earlier attendance in the first years of life (ISTAT 2020). Therefore, many infants are already engaged in interactions with peers and adults beyond their primary caregivers.

**TABLE 1:** Participants' characteristics.

		*n*	%
Age mean (± SD)	Infants	254 days (± 9.01 days)	
	Caregivers	35.9 years (± 4.42 years)	
Order of birth	First born	52	88.14
	Second born	7	11.86
Ethnicity	European	57	96.61
	Latina‐European	1	1.69
	Latina	1	1.69
Other language spoken at home	None	46	77.97
	English	4	6.78
	Greek	1	1.69
	Portuguese	1	1.69
	Polish	1	1.69
	Catalan	1	1.69
	Russian	1	1.69
	German	1	1.69
	Arabic	1	1.69
	Spanish and English	2	3.39

Fifty‐six participants, equally assigned to the experimental conditions, provided data for the HRV analysis. Three infants (*N* = 2 in the inclusion condition and *N* = 1 in the exclusion condition) were excluded from the HRV analysis because they had a percentage of beats corrected via Kubios > 5%. This threshold was applied because an excessive number of corrected beats can cause significant distortion of HRV analysis results, including suppressed variability; it is therefore recommended that corrected beats remain below 5% of the total (Tarvainen et al. 2021).

Fifty‐two participants provided data for the caregiver‐infant interaction analysis. Thus, four more infants (*N* = 2 in the inclusion condition and *N* = 2 in the exclusion condition) were discarded from the caregiver‐infant interaction coding due to technical issues. The final sample size was in line with the a priori calculation for a 2 × 2 repeated measures ANOVA, done via G*Power 3.1 (Faul et al. 2007), which required 52 participants with *α* = 0.05 and power = 0.80 to detect a medium effect (*f* = 0.20), and in accord with the a priori calculation of the estimated sample size for a 3 × 2 repeated measures ANOVA, which required 42 participants with *α* = 0.05 and power = 0.80 to detect a medium effect (*f* = 0.20).

The protocol was carried out in accordance with the ethical standards of the Declaration of Helsinki (BMJ 1991; 302: 1194) and approved by the ethical committee of the University of Milano‐Bicocca (Protocol number: 813). Data collection took place at the Bicocca Child & Baby Lab of the University of Milano‐Bicocca, from March 2024 to May 2025. Participants were recruited via phone calls and/or emails sent to parents based on birth records provided by the Metropolitan city of Milan and some neighboring municipalities. Only infants with informed written consent and privacy forms completed and signed by their parents were tested, in line with European and Italian regulations on personal data and images/video recording acquisition and processing.

### 2.2 Procedure

All participants were tested individually at the Bicocca Child & Baby Lab. Each infant was accompanied by a caregiver and took part in an experimental session consisting of three steps (Figure 1). In the first place, three electrocardiogram (ECG) electrodes were placed on the infant's chest to record heart rate throughout the session. The infant and caregiver then engaged in a free‐play period of about 2 min, during which the infant sat on the parent's lap facing a table with age‐appropriate toys on it. In the second place, infants remained seated at the same table while two experimenters sat in front of them. Participants took part in a face‐to‐face triadic ball‐tossing game aimed at manipulating social exclusion and were pseudo‐randomly assigned to either the inclusion or exclusion condition (as in Quadrelli et al. 2023, 2025). Both the free‐play and ball‐tossing task were video‐recorded for offline coding. Lastly, we administered to every infant the Cognitive Scale of Bayley Scales of Infant and Toddler Development‐Third Edition (Bayley‐III; Bayley 2006). All participants included in our final sample fell within a percentile range from a minimum of 16 to a maximum of 84 (*M* = 45.8 and SD = 16.9), indicating that the cognitive development of our participants was within the expected limits for their age.

At the end of the session, a brief play period was included to ensure the infant felt re‐included and left the lab in a positive mood. Caregivers were debriefed and had the opportunity to ask questions.

#### 2.2.1 Caregiver‐Infant Interaction

In the first phase of the study, after the three ECG electrodes were placed on the infant's chest to record infants' electrocardiogram for the entire duration of the session, the caregiver‐infant dyad was involved in a free‐play phase of about 2 min (Gartstein et al. 2021) aimed at assessing the quality of caregiver‐infant interaction and facilitating the infant's adaptation to the experimental setting. This duration of the interaction was chosen for two main reasons. First, it was constrained by the overall study protocol: given that the study's primary focus was the experimental ball‐tossing game manipulation, the free‐play segment was designed to be brief to accommodate the attentional capacity of 8‐month‐old infants. Second, this segment also served as an ECG recording in a neutral condition prior to the experimental manipulation (i.e., the ball‐tossing game). A 2‐min duration has been previously used to assess Sensitivity, Synchrony (e.g., Gartstein et al. 2018, 2021) and Mind‐Mindedness (Bigelow et al. 2015), although it falls within the lower end of the range typically reported in the literature.

The infant was seated on the caregiver's lap facing a desk on which a box of assorted toys (e.g., books, blocks, stuffed animals) was provided. The dyad was left alone and instructed to play “as they would normally do at home” making free use of the available materials. The free‐play interaction was video‐recorded to allow subsequent coding.

#### 2.2.2 Interaction Dynamics and Mind‐Mindedness Coding

Caregiver‐infant interaction dynamics were analyzed using the same coding scheme used by Gartstein et al. (2021). Our aim was to assess whether parental Sensitivity and dyad Synchrony were linked to infant vagal tone both during the free‐play phase and during the experimental manipulation (i.e., face‐to‐face ball tossing game). Specifically, we focused on two dimensions: (1) Sensitivity/Responsiveness, referring to the caregiver's promptness, contingency, warmth, and support in responding to the child, as well as the accurate interpretation of the child's needs and communication; (2) Reciprocity/Synchrony, referring to the simultaneity, similarity, coordination and fluidity of movements within the dyad. These two indexes were selected because it has been shown that they are related to infant vagal tone and emotion regulation (Puglisi et al. 2023; Rattaz et al. 2023). Both dimensions were coded separately in accordance with Gartstein et al. (2021), using a 7‐point Likert scale, where 1 indicated extremely non‐responsive/non‐reciprocal behavior and 7 indicated extremely responsive/reciprocal behavior. A research assistant, who was unaware of the hypotheses of the study, coded for parental Sensitivity and dyad Synchrony. Approximately 40% (*N* = 18) of the videos were additionally and independently coded for reliability by two additional raters, who were blind to the study's hypotheses. Intraclass correlation coefficients (ICCs) were calculated via R statistical software (R Core Team 2023, version 4.3.2), employing “irr” package (Gamer et al. 2019) and found to be excellent for both indexes, with values ranging from 0.917 to 0.984 and a mean ICC of 0.954 (all *ps* < 0.001).

In addition, we assessed parental Mind‐Mindedness (Meins and Fernyhough 2015). Mind‐Mindedness reflects the caregiver's tendency to treat the infant as an individual with a mind, interpreting the child's behavior in terms of their internal states. Caregivers' verbalizations were transcribed verbatim and segmented into individual comments based on temporal or semantic discontinuities (Meins and Fernyhough 2015). Caregiver's comments were coded as mind‐related if they used explicit terms referring to the child's internal state to describe what the child might be feeling, experiencing, or thinking, or if the caregiver spoke on behalf of the child. All other utterances were coded as non‐mind‐related. Mind‐related comments could be further coded as attuned or non‐attuned to the infant. A comment is considered attuned to the infant if the coder agrees with the caregiver's interpretation of the infant's internal state, connects the present activity to past or future experiences, or helps the interaction continue by providing cues when the infant pauses or seems unsure what to do next (Meins and Fernyhough 2015). The percentage of each category was calculated by dividing the number of mind‐related and attuned mind‐related comments by the total number of comments made by the caregiver, and multiplying by 100. For our analysis we considered the percentage of attuned mind‐related comments as it was shown that, during the first year of life, appropriate mind‐related comments are associated with infant physiological emotion regulation (Zeegers et al. 2018). A research assistant, who was unaware of the hypotheses of the study, coded for parental Mind‐Mindedness. Approximately 40% (*N* = 19) of the videos were additionally and independently coded for reliability by two additional raters, who were blind to the study's hypotheses. ICC was calculated via R statistical software (R Core Team 2023, version 4.3.2), employing “irr” package (Gamer et al. 2019) and found to be good for the attuned mind‐related comments, with an ICC of 0.993, *p* < 0.001.

#### 2.2.3 Face‐to‐Face Ball‐Tossing Game

The face‐to‐face ball‐tossing game was conducted with all participants seated around the table. The infant, staying on the caregiver's lap as in the free‐play interaction, and the two experimenters seated opposite. Our study followed the same procedure adopted by Quadrelli et al. (2023, 2025) and participants were pseudo‐randomly assigned to either the inclusion or exclusion condition. The game comprised a total of 18 ball‐tosses. The first six throws were equally distributed between participants in both conditions, with the experimenters smiling and interacting with the infant. However, the last 12 throws were divided as follows: in the inclusion condition, the infant continued to receive and throw the ball ensuring fair participation; while in the exclusion condition, the participant was systematically ignored, with the experimenters continuing the game exclusively between themselves (Quadrelli et al. 2023, 2025).

Parents were instructed to remain neutral and not to interact with the infant throughout the game. If the infant needed help to throw the ball, the parent could intervene, without talking. We verified that, when accounting for the proportion of throws performed by each infant in each condition (i.e., inclusion vs. exclusion), there were no significant differences in the percentage of times excluded infants were helped compared to included ones. In other words, even if excluded infants made fewer throws than included ones, the proportion of throws that resulted in help did not differ between conditions, as confirmed by an independent‐samples *t*‐test, yielding a non‐significant result, *t*(57) = −1.62, *p* = 0.112, *d* = −0.421.

Infants' ECG was continuously registered, and sessions were video‐recorded for behavioral analysis. Recordings began with the first movement initiating the game and ended with the final reception of the last throw. For the purposes of our study, and as done by Quadrelli et al. (2023, 2025), the video recordings were divided as follows: (a) baseline phase, which comprised the first six throws of the ball; (b) experimental phase, that includes the last twelve tosses, and which differentiates the two experimental conditions (i.e., inclusion and exclusion).

#### 2.2.4 Behavioral Responses Coding

Following the coding scheme of Quadrelli et al. (2023, 2025), all videos were segmented into two‐second intervals for offline coding. Four composite indices were derived by summing the behavioral scores.

1. Positive Emotionality: including smiling, happy vocalizations, and leaning forward toward other players.
2. Negative Emotionality: consisting of crying, angry vocalizations, negative facial expressions, slumped posture, withdrawal from the game, and seeking help from the parent.
3. Active Engagement: comprising raising arms to request the ball, pointing toward the ball, and seeking the player's attention (e.g., striking the table with their hands).
4. Visual Attention: including looking at the ball and attending to the other players.

All behaviors were coded as present (1) or absent (0) within each segment. For both baseline and experimental phases, behavioral scores were summed and then normalized by dividing the total by the number of observed behaviors. The resulting values were multiplied by 100 to obtain percentage scores. A research assistant, who was unaware of the hypotheses of the study, coded for infants' behavior during the ball‐tossing game. Approximately 30% (*N* = 16) of the videos were additionally and independently coded for reliability by one additional rater, who was blind to the study's hypotheses. Intraclass correlation coefficients (ICCs) were calculated via R statistical software (R Core Team 2023, version 4.3.2), employing “irr” package (Gamer et al. 2019) and found to be excellent for all indexes in the two phases, with values ranging from 0.95 to 1 and a mean ICC of 0.991 (all *ps* < 0.001).

#### 2.2.5 Electrophysiological Data Recording and Processing

Infant's electrocardiogram (ECG) was recorded during both free‐play and triadic ball‐tossing game using the ProComp Infiniti (Thought Technology, Montreal, QC, Canada), a multimodal physiological monitoring device enabling real‐time acquisition of biological signals. ECG recordings were obtained with an ECG‐Flex/Pro sensor connected to three disposable electrodes. Signals were recorded continuously and stored for offline analysis. After initial visual inspection, the data were segmented according to experimental phases using BioGraph Infiniti (Thought Technology, Montreal, QC, Canada). Inter‐beat intervals (IBIs) were extracted with a sample rate of 256 Hz (Della Longa et al. 2021). Artifacts potentially affecting heart rate variability (HRV) measures were corrected using Kubios HRV Scientific Lite 4.1.0 (Kubios Oy, Kuopio, Finland). Anomalous IBIs were identified via a threshold‐based correction using a “Very Low” threshold. This approach preserves physiological beat‐to‐beat fluctuations typical of developmental samples while ensuring accurate HRV estimates (Alcantara et al. 2020).

Corrected data were used to calculate the Root Mean Square of Successive Differences (RMSSD), and index of HRV (Shaffer and Ginsberg 2017), for each experimental phase. RMSSD reflects short‐term beat‐to‐beat variability and vagal influence on cardiac rhythm (Pham et al. 2021) and is computed by determining the time differences between successive heartbeats in milliseconds, squaring each value, averaging them, and then taking the square root of the result (Shaffer and Ginsberg 2017). Among the time domain methods used to assess vagal influence on HRV, RMSSD is one of the most common indexes.

## 3 Data Analysis

All statistical analyses were conducted using Jamovi 2.2.5 (https://jamovi.org) using a two‐tailed 0.05 level of significance.

Preliminary analyses were conducted to examine whether background variables were associated with the main study variables. First, we tested whether infants' sex interacted with the main analyses. No significant effects were found for either the RMSSD analyses (all *ps* > 0.50) or the behavioral reactivity analyses (all *ps* > 0.054). Second, we examined whether parental age was associated with caregiving interaction variables. Correlation analyses revealed no significant associations between parental age and parental sensitivity, synchrony, or mind‐attuned comments (all *ps* > 0.18). Finally, we tested whether differences in interaction dynamics emerged as a function of infants' or caregivers' sex. No significant differences were found for either caregivers' or infants' behaviors (all *ps* > 0.098). Given the absence of significant associations, these background variables were not included as covariates in the main analyses.

First, to assess whether physiological activation variations occurred between conditions in the three phases of the procedure, and to assess whether excluded infants exhibited more physiological stress than their included peers, we conducted a 3 × 2 repeated measures ANOVA with RMSSD as dependent variable, Phase as within subject variable (i.e., free‐play, baseline phase, experimental phase) and Condition as between subject variable (i.e., inclusion, exclusion).

Secondly, to test whether infants displayed a different modulation of behavioral and emotional reactivity in the exclusion and inclusion conditions, and thus to assess whether we could replicate results with 13‐month‐old infants by Quadrelli et al. (2023, 2025), multiple 2 × 2 repeated measures ANOVAs were conducted, with Condition as between subject factor (inclusion vs. exclusion) and Phase as within subject factor (baseline phase vs. experimental phase). Four ANOVAs were conducted, one for each behavioral index coded (i.e., Positive Emotionality, Negative Emotionality, Active Engagement and Visual Attention).

Lastly, to explore the possible relations between parent‐child interaction dynamics, Mind‐Mindedness, and infant RMSSD across the three phases of the procedure, linear regression analyses were conducted. Given the potential overlap among predictors, we first examined Spearman's correlations among Sensitivity, Synchrony, and Mind‐Mindedness. Based on the correlation structure, predictors were grouped accordingly. Since Sensitivity and Synchrony significantly correlated with each other, *ρ*(50) = 0.687, *p* < 0.001, they were entered simultaneously in a multiple regression model. As Mind‐Mindedness did not significantly correlate with the others (all *ps* > 0.150), it was entered in a separate simple linear regression. This approach was applied for each phase of the study: the free‐play phase, the baseline phase, and the experimental phase (inclusion and exclusion conditions separately), yielding a total of eight regression models. The dependent variable in each model was infant RMSSD during the corresponding phase. Given that these are secondary and exploratory analyses, no multiple comparison adjustments were performed (Bender and Lange 2001; Lee et al. 2023).

For all analyses, when the ANOVAs yielded significant effects, Post‐Hoc tests were conducted and Holm‐Bonferroni correction was used (Abdi 2010). The data are reported as means and standard errors (SEs). See Supporting Information S1 for results tables.

## 4 Results

### 4.1 Infant's Heart Rate Variability (RMSSD)

To assess whether included and excluded infants exhibited differences in their RMSSD variations throughout the experimental procedure, and especially in the experimental phase of the ball‐tossing game, a 3 × 2 repeated measures ANOVA was conducted, with Phase (free‐play, baseline phase, experimental phase) as within subject factor, Condition (inclusion, exclusion) as between subjects factor and RMSSD values as dependent variable.

**FIGURE 2:**
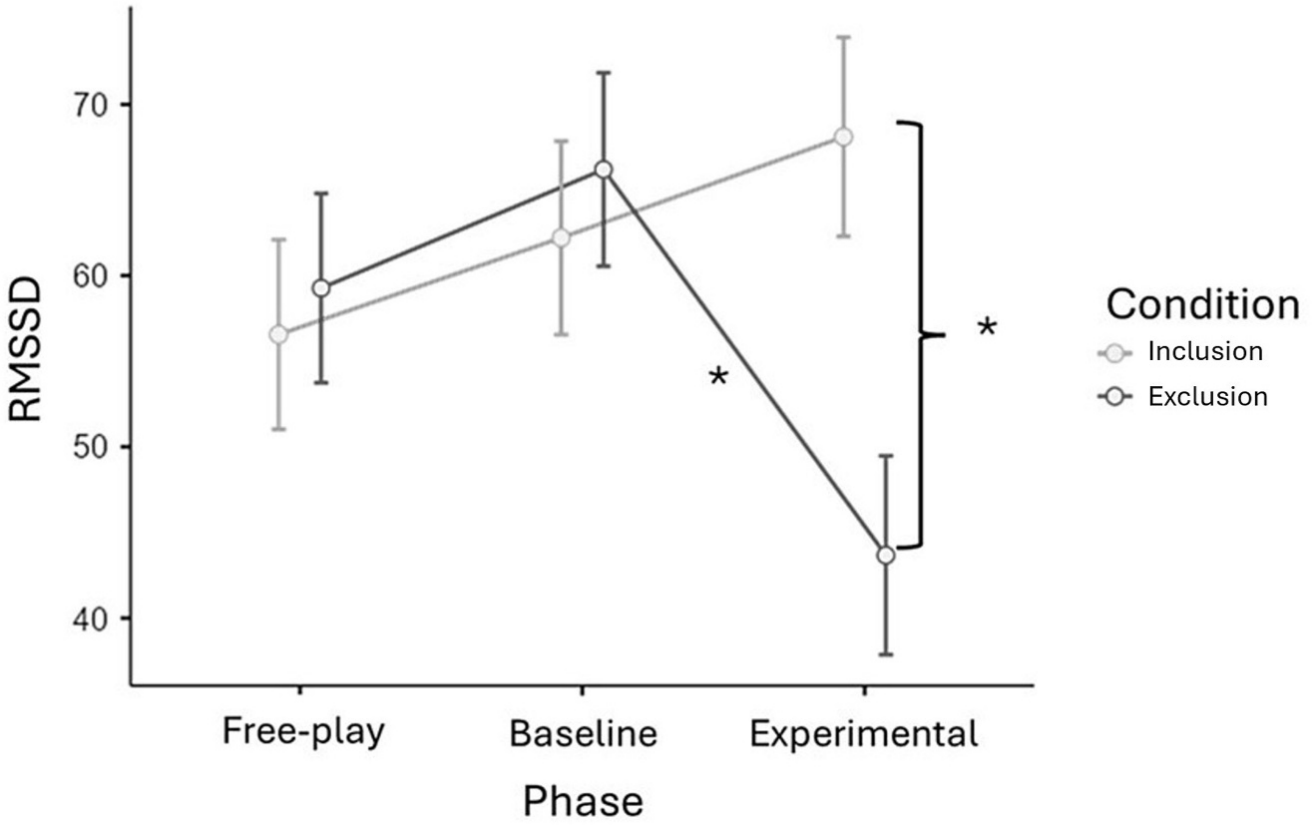
Infants' RMSSD (Root Mean Square of Successive Differences) as a function of Phase (free‐play, baseline, experimental) and Condition (inclusion, exclusion). Error bars represent standard errors (SEs) and significant differences between means are indicated by an asterisk, *p* < 0.05.

Results showed a statistically significant interaction between Phase and Condition, *F*(2, 108) = 8.57, *p* < 0.001, *η* ^2^_*p*_ = 0.137. Post‐Hoc tests revealed a significant reduction in RMSSD values in the experimental phase (*M* = 43.7 ms, SE = 5.81 ms) compared to the baseline phase (*M* = 66.2 ms, SE = 5.65 ms) in the exclusion condition, *t*(54) = 4.256, *p* < 0.001. This suggests greater physiological activation associated with stress response in excluded infants. Moreover, comparing the two conditions in the experimental phase, included participants exhibited significantly higher RMSSD values (*M* = 68.1 ms, SE = 5.81 ms) than excluded ones, *t*(54) = 2.973, *p* = 0.032, indicating a more relaxed physiological state in included participants compared to excluded ones (Figure 2). No other significant comparison or main effect was found, all *ps* > 0.07. Therefore, these analyses indicate that no difference between participants was found either in the free‐play phase or during the baseline phase of the ball tossing game, or between these phases, suggesting that both groups had a similar physiological activation before experiencing the experimental manipulation (i.e., experimental phase). See Supporting Information S1: Table S1 and S2 for results tables.

### 4.2 Face‐to‐Face Ball‐Tossing Game Responses Coding

#### 4.2.1 Positive Emotionality

**FIGURE 3:**
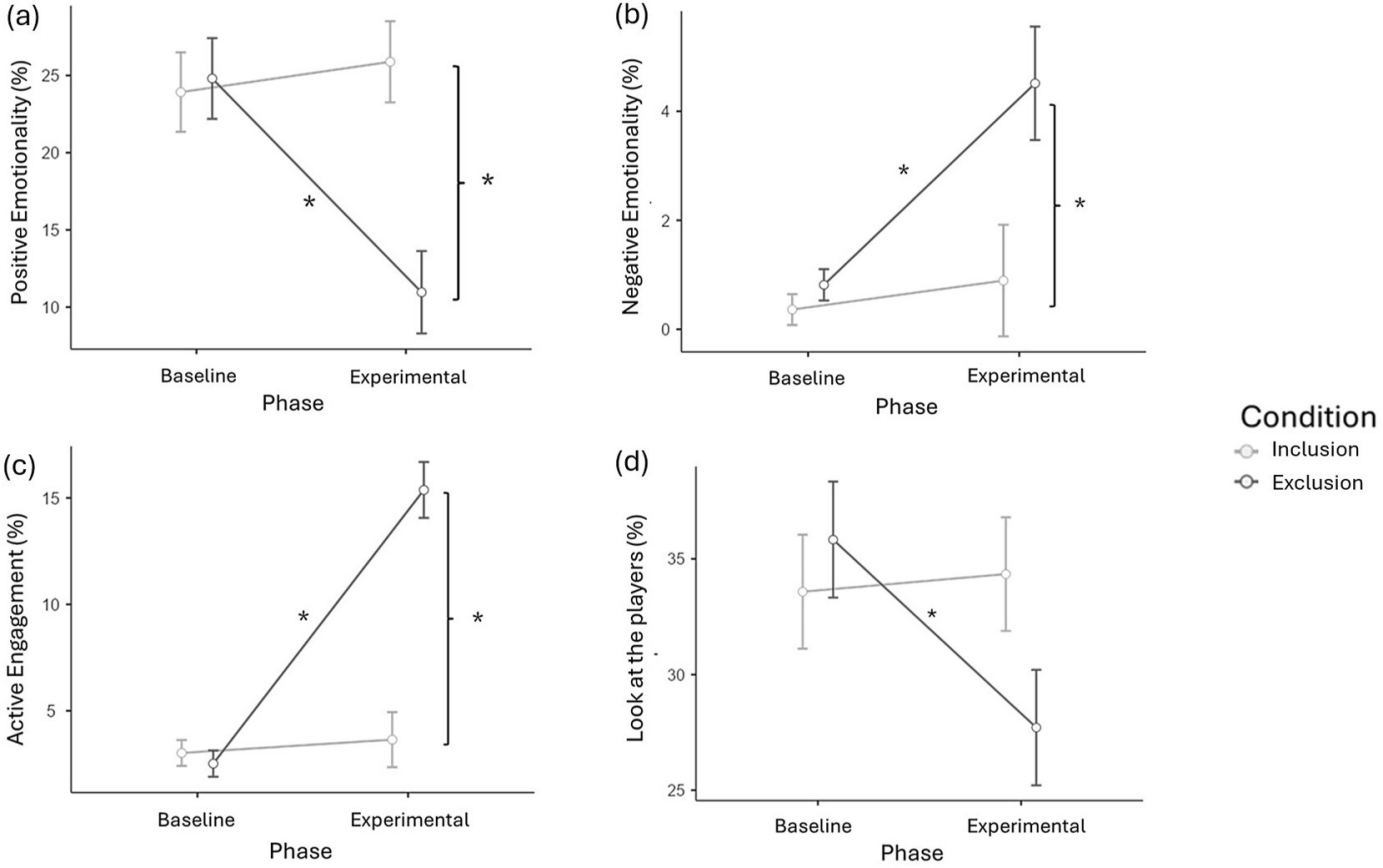
Infants' (a) Positive Emotionality, (b) Negative Emotionality, (c) Active Engagement, and (d) Look at the players during the triadic ball‐tossing game as a function of Phase (baseline, experimental) and Condition (inclusion, exclusion). Error bars represent standard errors (SEs) and significant differences between means are indicated by an asterisk, *p* < 0.05.

A repeated measure ANOVA was conducted to assess whether being included versus excluded could affect infants' Positive Emotionality and whether this was especially true in the experimental phase, where the throws are equally distributed between players in the inclusion condition and only between the two experimenters in the exclusion condition. Results showed a main effect of Phase *F*(1, 57) = 16.9, *p* < 0.001, *η* ^2^_*p*_ = 0.228, with infants expressing more Positive Emotionality during the baseline phase (*M* = 24.4%, SE = 1.83%) than the experimental phase (*M* = 18.4%, SE = 1.87%). In addition, we found a main effect of Condition, *F*(1, 57) = 4.24, *p* = 0.044, *η* ^2^_*p*_ = 0.069, with excluded infants showing lower percentage scores (*M* = 17.9%, SE = 2.43%) than included ones (*M* = 24.9%, SE = 2.39%). Finally, we identified an interaction effect between Phase and Condition, *F*(1, 57) = 29.8, *p* < 0.001, *η* ^2^_*p*_ = 0.344. Post‐Hoc analysis showed that excluded infants exhibited a significant decrease in Positive Emotionality from baseline (*M* = 24.8%, SE = 2.62%) to the experimental phase (*M* = 11.0%, SE = 2.67%), *t*(57) = 6.709, *p* < 0.001. Furthermore, a significant difference was also found, *t*(57) = 3.989, *p* = 0.001, between the two groups in the experimental phase: excluded children displayed significantly fewer positive behaviors compared to included children (*M* = 25.9%, SE = 2.62%) (Figure 3). No other comparison resulted significant, all *ps* > 0.67.

#### 4.2.2 Negative Emotionality

A repeated measure ANOVA was conducted to assess whether being included versus excluded could affect infants' Negative Emotionality and whether this was especially true in the experimental phase, where we could expect an increase of this behavior in those infants excluded from the ball‐tossing game. Results revealed a statistically significant main effect of Phase, *F*(1, 57) = 10.60, *p* = 0.002, *η* ^2^_*p*_ = 0.157, indicating an increase in behaviors expressing Negative Emotionality from the baseline (*M* = 0.59%, SE = 0.20%) to the experimental phase (*M* = 2.70%, SE = 0.73%). A significant main effect of Condition was also found, *F*(1, 57) = 5.71, *p* = 0.020, *η* ^2^_*p*_ = 0.091, showing that children in the excluded group displayed more negative behaviors (*M* = 2.66%, SE = 0.61%) compared to included children (*M* = 0.63%, SE = 0.60%). Results also revealed a significant interaction between Condition and Phase, *F*(1, 57) = 5.94, *p* = 0.018, *η* ^2^_*p*_ = 0.094. Specifically, a Post‐Hoc test showed that in the experimental phase, behaviors reflecting Negative Emotionality were significantly higher in the exclusion condition (*M* = 4.51%, SE = 1.04%) compared to the inclusion condition (*M* = 0.89%, SE = 1.02%), with a significant difference, *t*(57) = −2.48, *p* = 0.049. Moreover, comparing the exclusion condition between the baseline phase (*M* = 0.82%, SE = 0.29%) and the experimental phase, an increase in such behaviors was observed in the latter phase, with a significant difference, *t*(57) = −3.99, *p* = 0.001 (Figure 3). No other significant difference was found, all *ps* > 0.53.

#### 4.2.3 Active Engagement

A repeated measure ANOVA was conducted to investigate whether, during the ball tossing game and especially in the experimental phase, differences in Active Engagement could be detected between included and excluded infants. A significant main effect of Phase was found, *F*(1, 57) = 54.3, *p* < 0.001, *η* ^2^_*p*_ = 0.488, indicating that behaviors within the cluster were significantly higher in the experimental phase (*M* = 9.51%, SE = 0.92%) compared to the baseline phase (*M* = 2.77%, SE = 0.43%). Analyses also revealed a statistically significant main effect of Condition, *F*(1, 57) = 25.5, *p* < 0.001, *η* ^2^_*p*_ = 0.309, highlighting that excluded infants (*M* = 8.94%, SE = 0.79%) exhibited significantly higher levels of requesting the ball and seeking the player's attention compared to included children (*M* = 3.33%, SE = 0.78%). Finally, a significant interaction between Phase and Condition was observed, *F*(1, 57) = 44.7, *p* < 0.001, *η* ^2^_*p*_ = 0.440. Post‐Hoc comparisons revealed that excluded children showed a significant increase in Active Engagement behaviors from the baseline phase (*M* = 2.52%, SE = 0.62%) to the experimental phase (*M* = 15.37%, SE = 1.31%), *t*(57) = −9.86, *p* < 0.001. Moreover, a significant difference, *t*(57) = −6.37, *p* < 0.001, was also found between the two groups in the experimental phase (Figure 3): excluded participants showed greater active involvement compared to included participants (*M* = 3.64%, SE = 1.29%). No other significant comparison was found, all *ps* > 0.99.

#### 4.2.4 Visual Attention

To test whether excluded infants could display more Visual Attention to the ball‐tossing game than included ones a repeated measures ANOVA was conducted. No significant effect emerged, all *ps* > 0.125. Therefore, to explore such results, we decided to split our analysis into two ANOVAs with Phase and Condition as independent variables and “looking at the ball” or “looking at the players” as dependent variables, as it was done in previous studies which have considered these two behaviors separately (Testa et al. 2025). In addition, Quadrelli et al. (2023) had analyzed these behaviors separately in the Supplementary Materials section of their work, highlighting possible interesting considerations that might shed light on our lack of results for the Visual Attention cluster.

Regarding “looking at the ball”, a significant interaction between Phase and Condition was found, *F*(1, 57) = 8.19, *p* = 0.006, *η* ^2^_*p*_ = 0.126; however, Post‐Hoc comparisons did not reveal any significant differences, all *ps* > 0.092. In contrast, for the behavior “looking at the players”, the ANOVA revealed a significant main effect of Phase, *F*(1, 57) = 5.26, *p* = 0.026, *η* ^2^_*p*_ = 0.084, with more looks toward players in the baseline phase (*M* = 34.7%, SE = 1.76%) compared to the experimental phase (*M* = 31.0%, SE = 1.75%). Also, a significant interaction between Phase and Condition also emerged (Figure 3), *F*(1, 57) = 7.66, *p* = 0.008, *η* ^2^_*p*_ = 0.118. Specifically, in the exclusion condition, looks toward players significantly decreased from the baseline phase (*M* = 35.8%, SE = 2.51%) to the experimental phase (*M* = 27.7%, SE = 2.50%), with a significant difference, *t*(57) = 3.55, *p* = 0.003. All other *ps* > 0.19.

Results about behavioral reactivity are also presented in Supporting Information S1: Tables S3 and S4.

### 4.3 Exploratory Associations Between Parent‐Child Interaction Dynamics, Mind‐Mindedness, and RMSSD

To investigate whether parental sensitivity, dyadic synchrony, and caregiver's mind‐attuned comments predicted infant RMSSD across the three phases of the procedure, we conducted exploratory linear regressions. Given that Sensitivity and Synchrony correlated with each other, they were entered in a multiple regression model, while Mind‐Mindedness was tested in a separate simple regression.

We first started exploring the effects of dyads interaction dynamic and parental Mind‐Mindedness on the RMSSD during the free‐play condition. We found that only attuned mind‐related comments predicted significantly infant RMSSD variations *F*(1, 50) = 4.29, *p* = 0.044, *R* ^2^ = 0.079. Indeed, a higher percentage of comments was associated with lower RMSSD, *β* = −0.281, *t*(50) = −2.07. To explore whether Sensitivity, Synchrony, and Mind‐Mindedness predict infant RMSSD during the ball‐tossing game, we examined their associations with RMSSD both in the baseline phase and in the experimental phase, considering included and excluded infants separately in the latter. No significant associations were found in the baseline phase, all *ps* > 0.109. However, we did find that, in the exclusion condition, during the experimental phase Synchrony significantly predicted infant's RMSSD, *F*(1,23) = 5.39, *p* = 0.029, *R* ^2^ = 0.190, with higher values of Synchrony associated with lower RMSSD, *β* = −0.789, *t*(23) = −2.32. All other *ps* > 0.063. These results are also presented in Supporting Information S1: Tables S5 and S6.

## 5 Discussion

Available research has highlighted the fundamental human need to belong (Baumeister and Leary 1995) and evidenced that even in early infancy children display physiological and behavioral stress responses when social interactions are abruptly interrupted (Bazhenova et al. 2001; Haley and Stansbury 2003; Moore and Calkins 2004; Quadrelli et al. 2023, 2025). However, it remains unclear whether and how infants' physiological reactivity is specifically modulated by experiences of social exclusion, a distinct form of social disruption. Notably, it was shown that being excluded from a triadic social interaction has an impact on the physiological regulation in adults (Eres et al. 2021; Iffland et al. 2014; Kulakova et al. 2024).

In the present research we investigated whether being included or excluded during a live triadic ball‐tossing game impacts 8‐month‐olds’ physiological and behavioral reactivity during the game. In addition, we explored if parental mind attuned comments and interaction dynamics are related to infant's physiological regulation in this context. In line with previous studies (Basset et al. 2025; Quadrelli et al. 2023, 2025; Testa et al. 2025), we divided the ball‐tossing game into two phases: baseline (first six throws) and experimental (last twelve throws). This design allowed us to examine changes in behavioral reactivity and to test whether infant HRV, indexed by RMSSD, was influenced by the phase of the game. Before the ball‐tossing task, we also included a brief parent‐infant free‐play session to collect RMSSD data prior to the triadic interaction and to observe and code the dyad's interaction dynamics.

### 5.1 Physiological Reactivity

Results from physiological reactivity analysis showed no significant differences in RMSSD between the free‐play and baseline phases across both groups, suggesting that infants maintained a comparable level of physiological activation in these contexts. Similarly, no differences emerged between the baseline and experimental phases for included infants, indicating stable vagal tone throughout the game and reflecting a relaxed autonomic state (Oliveira et al. 2019). This stability suggests that, in the absence of social exclusion, infants were able to regulate their physiological activity consistently across the interaction. In other words, when social exchanges remain predictable and supportive, infants can maintain a balanced autonomic response even as the social demands of the task evolve (i.e., such as shifting from a dyadic to a triadic interaction), which may serve as a protective factor fostering adaptive engagement with the environment.

In contrast, excluded infants displayed a significant decrease in RMSSD from baseline to the experimental phase, suggesting a potentially stressful context for infants. This finding aligns with evidence that HRV is sensitive to autonomic nervous system changes under stress (Kim et al. 2018). In particular, previous research has shown that infants typically exhibit reduced HRV when shifting from free‐play to stressful conditions, such as maternal still‐face (Bazhenova et al. 2001), supporting the view that lower HRV is generally associated with stress reactivity (Pulopulos et al. 2018). Our results also converge with adult studies reporting cardiac responses to ostracism (e.g., Eres et al. 2021; Iffland et al. 2014) and extend these findings to a preverbal sample. Importantly, we also observed a significant difference in RMSSD during the experimental phase between included and excluded infants, a result consistent with social exclusion eliciting a stress response and distinct patterns of physiological activation.

Overall, these results suggest that, already at a very early age, being excluded from a social interaction elicits a measurable stress response. These data can be framed within the theoretical perspective of the Polyvagal Theory (Porges 2001), according to which a decrease in vagal tone reflects a transition from a state of safe social regulation to a more primitive defensive response, triggered in conditions perceived as threatening. This is in fact the case of social exclusion, an experience that threatens fundamental human needs such as the need to belong (Williams 2007). Taken together, these findings highlight the importance of integrating physiological measures, such as RMSSD, when studying early social development. While RMSSD provides a sensitive index of autonomic regulation, it is through its combination with observable behaviors that we can gain a more comprehensive understanding of how infants respond to inclusion and exclusion experiences. This multimethod approach is particularly relevant in early infancy, when verbal reports are not yet available and both physiological and behavioral data are essential to capture the complexity of infants' adaptive responses.

### 5.2 Behavioral Reactivity

Our physiological findings align with the behavioral patterns we observed: excluded infants showed reduced Positive Emotionality, increased Negative Emotionality, and higher Active Engagement (Quadrelli et al. 2023, 2025), with significant differences from included infants during the experimental phase across all these behavioral categories. Indeed, infants, during the experimental condition, showed more Positive Emotionality when included as compared to when excluded. This finding aligns with previous studies showing that infants at 13 months (Quadrelli et al. 2023, 2025), 3–5‐year‐old children (Testa et al. 2025), 10‐year‐old children (Basset et al. 2025) and adults (Williams and Sommer 1997) show greater positive emotions (such as, e.g., smiling and/or laughing) when included rather than ostracized, drawing a developmental trajectory that seems to remain stable across ages. Regarding Negative Emotionality, our results are in line with these earlier studies, suggesting that as early as 8 months infants respond differentially to being excluded from a triadic ball‐tossing game by exhibiting more negative emotionality, as older children and adults, showing a great consistency across developmental stages. This result, in particular, is consistent with the patterns we observed for RMSSD, showing reduced vagal activity during exclusion. We interpret these findings as reflecting infants' response to social exclusion. However, an alternative explanation is that infants' response may be driven by the loss of a desired toy rather than by social exclusion itself. This interpretation cannot be ruled out based on the present data and should be directly tested in future studies. In the present paradigm the ball functions not only as a desirable object, but also as a medium of social interaction, as it is embedded in a turn‐taking exchange with social partners. Thus, reduced access to the ball also entails reduced participation in a shared social activity. Moreover, infants were first exposed to an inclusion phase (i.e., baseline phase), which may have established expectations both of reciprocal interaction and of continued access to the ball. The subsequent interruption of this pattern in the exclusion condition may therefore constitute a violation of infants' expectations regarding the social interaction, their expectation of receiving the ball, or both. The present paradigm does not allow these possibilities to be disentangled. At the same time, the pattern of results on Active Engagement provides some support for a social interpretation. Specifically, excluded infants not only requested the ball more frequently, but also directed more bids toward the experimenters, particularly during the experimental phase, compared to included infants. This pattern is consistent with previous findings (Quadrelli et al. 2023) and suggests that infants' responses may not be limited to object‐directed frustration, but may also involve increased attempts to re‐engage socially. Consistent with this interpretation, previous research has shown that humans often adjust their behavior to regain social affiliation (e.g., Marinović et al. 2017; Over and Carpenter 2009). Importantly, we interpret these findings with caution. While they are consistent with the possibility that infants are sensitive to disruptions in social interaction, they do not allow us to conclude that infants experience social exclusion in the same way as older individuals, as their subjective experience cannot be directly assessed. Rather, our results indicate that infants show heightened distress and engagement behaviors when excluded from an ongoing interaction, which we interpret as evidence of an early sensitivity to social contingencies.

Finally, we did not find any significant difference for the Visual Attention cluster. However, in the context of social exclusion, and given the young age of our participants, we thought it may be more informative to consider the two behaviors composing this cluster (i.e., percentages of gazes toward the ball and toward the players) separately. Our choice was also based on previous evidence indicating that children in the exclusion condition direct greater attention to social partners during social exchanges (Quadrelli et al. 2023; Testa et al. 2025). Based on these premises we showed that in our sample there were no significant differences between included and excluded infants for “looking at the ball” behavior, but we could highlight some differences for the percentages of gazes toward the players. In fact, excluded infants reduced their gaze toward other social partners from baseline to the experimental phase. However, even if this result is in contrast with previous studies (Quadrelli et al. 2023; Testa et al. 2025), it is in line with other research suggesting that social withdrawal represents a possible reaction to social exclusion (Büttner et al. 2024; Lei et al. 2024; Syrjämäki et al. 2020). Thus, a decrease in looking at others might reflect a defensive strategy aimed at coping with the stress of being excluded. In fact, during the first year of life, self‐regulation emerges through behaviors such as gaze aversion and self‐distraction (Field 1981). Indeed, disengagement of attention is widely used as a measure of self‐regulation (Frick et al. 2018).

Overall, our study shows that by 8 months, infants already show distinct behavioral responses depending on whether they are included or excluded in a triadic interaction, therefore replicating and extending the findings of Quadrelli et al. (2023) to an earlier developmental stage. This pattern is further supported by our RMSSD data, indicating that physiological regulation also varies according to social inclusion or exclusion. These results are consistent with the idea that a system sensitive to social exclusion may be active very early in life.

### 5.3 Interaction Dynamics and Parental Mind Attuned Comments

Finally, we explored the possible effects of parental mind attuned comments, sensitivity and synchrony on infants' RMSSD during all phases of the game. Our regression analyses revealed distinct patterns depending on the context and the type of parental behavior. During the free‐play condition, caregivers' mind‐attuned comments significantly predicted infants' RMSSD. At higher levels of comments, infants showed diminished RMSSD, which is usually linked to vagal withdrawal. Mind‐Mindedness refers to the parent's ability to recognize and comment on the child's mental states, such as thoughts, emotions, and desires (Meins and Fernyhough 2015). Studies have shown that mothers' appropriate mind related comments at 4 and 12 months predict higher baseline HRV at 12 months, suggesting more stable physiological regulation (Zeegers et al. 2018). In contrast, in our study we examined infants' immediate physiological reactivity during the moment the parent produced mind‐minded comments. At 8 months, processing these comments may require greater cognitive and emotional engagement, potentially increasing regulatory effort and explaining the observed association with lower RMSSD.

Lastly, during the ball‐tossing game, no significant associations emerged in the baseline phase, a result that seems to indicate that physiological activation before the experimental manipulation may be not systematically influenced by parental behaviors. However, in the experimental phase, when infants were excluded from the game, higher Synchrony predicted lower RMSSD values. Behavioral synchrony between parent and child is generally considered a marker of positive interaction (Puglisi et al. 2023). However, high levels of synchrony may also reflect greater physiological effort on the part of the child. For instance, physiological synchrony has been linked to increased reactivity in children of more anxious mothers, suggesting that synchrony may index regulatory effort rather than physiological calm (C. G. Smith et al. 2022). Consistent with this, Busuito et al. (2019) proposed that infants with greater regulatory needs may rely more heavily on synchrony with their caregiver to support self‐regulation. In this light, dyads characterized by higher synchrony during free play may be those in which infants already require greater co‐regulatory support, and may therefore show heightened physiological stress responses when that support becomes unavailable, as during social exclusion. However, given the exploratory nature of these analyses, and the small sample size involved, this topic warrants further investigation in future research, and these results need to be considered cautiously. Indeed, replication in a larger sample size will be necessary in order to be able to determine the robustness of these findings which may now be seen as a starting point for further in‐depth analyses. Overall, these exploratory results indicate that different aspects of parental behavior may play distinct roles in shaping infants' physiological responses depending on the social context.

### 5.4 Limitations and Future Directions

Some limitations of this study need to be acknowledged and addressed in future research. In the first place, there is the lack of post‐experimental assessment of behavioral and physiological responses, which prevents understanding the persistence or developmental impact of the observed effects. Future research could include follow‐up measures to examine recovery, compensation, and the temporal dynamics of social exclusion responses, as done in previous studies involving adult participants (Eres et al. 2021; Iffland et al. 2014; Kulakova et al. 2024). In addition, since it was shown that individual differences, such as non‐verbal intelligence, popularity and self‐esteem, moderate the effects of ostracism in older children (Tobia et al. 2017), the present study could have further examined infant individual characteristics, using additional measures that may help explain individual variability in responses to ostracism.

In the second place, another limitation concerns the blinding of coders to the experimental conditions during the ball‐tossing game. Although the condition was inherently visible in the video recordings, coders were blind to the specific aims and hypotheses of the study and were therefore unlikely to form systematic expectations about the expected results. Nevertheless, although our findings were consistent with those of previous studies (Quadrelli et al. 2023, 2025; Testa et al. 2026), the possibility that awareness of the condition may have unintentionally influenced the coding cannot be entirely ruled out. Future studies should consider alternative recording methods that would allow for fully blinded behavioral coding.

A further limitation concerns the duration of the free‐play segment used to assess interaction dynamics and Mind‐Mindedness. Although a 2‐min observation is consistent with previous studies (e.g., Bigelow et al. 2015; Gartstein et al. 2018, 2021), it represents a comparatively restricted observation period within the existing literature. Indeed, assessments of these constructs have been based on observation periods ranging from brief interactions to sessions lasting up to 20 min (Meins and Fernyhough 2015). Future research should consider using longer free‐play observations to provide a more comprehensive assessment of Mind‐Mindedness and interaction dynamics, particularly when these constructs are a primary variable of interest.

Moreover, although the sample size was consistent with the requirements indicated by the power analysis for our main analyses, future studies with larger samples are needed to more thoroughly investigate the interactions between relational dynamics, Mind‐Mindedness, and infants' physiological reactivity, which were explored on a preliminary basis in the present work. Additionally, the present sample was relatively homogeneous, which may limit the generalizability of the findings. Future studies should aim to recruit more heterogeneous samples in order to assess the robustness of these effects across different contexts. Finally, although pseudo‐random assignment was employed to balance experimental conditions, this approach does not guarantee full equivalence between groups, and potential residual differences between conditions should be considered when interpreting the results.

## 6 Conclusions

The present study suggests that, by 8 months of age, social exclusion affects infants' physiological reactivity and behavioral responses, potentially leading to negative cascading effects. These findings expand our understanding of how this phenomenon shapes early social and emotional development, extending previous research on preverbal populations (Quadrelli et al. 2023, 2025) to younger infants. Specifically, the results indicate that exclusion impacts both emotional and physiological regulation, suggesting that sensitivity to these interactional dynamics may emerge early in development. Given these negative impacts, the findings underscore the importance of early prevention and intervention to support typical social and emotional development. Future research may focus on how to promote inclusion in daycare environments and to support parents to foster their children's emotional regulation development.

## Author Contributions

**Giada Basset:** conceptualization, data curation, methodology, software, investigation, formal analysis, visualization, writing – original draft, writing – review and editing. **Ermanno Quadrelli:** conceptualization, methodology, formal analysis, funding acquisition, supervision, project administration, writing – review and editing. **Chiara Turati:** conceptualization, methodology, supervision, writing – review and editing. **Hermann Bulf:** conceptualization, methodology, supervision, funding acquisition, project administration, writing – review and editing.

## Funding

This work was supported by a grant from the Ministero dell’Università e della Ricerca ‐ NextGeneration EU (PNRR M4.C2.I1.1‐Avviso 104/2022, CUP H53D23004180006, Grant No. 2022‐NAZ‐0420) to Ermanno Quadrelli and by a grant from the Ministero dell'Università e della Ricerca ‐ NextGeneration EU (PNRR M4.C2.I1.1‐Avviso 104/2022, CUP H53D23004110006, Grant No. 2022‐NAZ‐0410) to Hermann Bulf. The funders had no role in study design, data collection and analysis, decision to publish, or preparation of the manuscript.

## Ethics Statement

The protocol was carried out in accordance with the ethical standards of the Declaration of Helsinki (BMJ 1991; 302: 1194) and approved by the ethical committee of the University of Milano‐Bicocca (Protocol number: 813).

## Conflicts of Interest

The authors declare no conflicts of interest.

## Supporting information


Supporting Information S1

## Data Availability

The data necessary to reproduce the analyses presented here are accessible at the following URL: https://osf.io/wr7jh/overview?view_only=991e2672f41a4f7e9f5e0dd0e358ed6c. The materials necessary to attempt to replicate the findings presented here are not publicly accessible, but available upon request to the first author. The analyses presented here were not preregistered.
