# Supplementary material for: The Stress of Being Left Out: Physiological and Behavioral Responses to Ostracism in Preverbal Infants
Source: Infancy. 2026 Sep 23;31(5):e70128. doi: 10.1111/infa.70128 (PMC13601642; doi:10.1111/infa.70128)
Supplement: Supplementary file 1 [file INFA-31-0-s001.docx]

**The stress of being left out: physiological and behavioral responses to ostracism in preverbal infants**

Supplementary materials

- Supplementary Table 1*. Physiological reactivity (RMSSD) ANOVA*
- Supplementary Table 2. *RMSSD Estimated Marginal Means*
- Supplementary Table 3. *Behavioral Reactivity ANOVAs*
- Supplementary Table 4. *Behavioral reactivity Estimated Marginal Means*
- Supplementary Table 5. *Linear regression between mind-attuned comments and infant’s RMSSD during the free-play phase*
- Supplementary Table 6. *Linear regression between Sensitivity, Synchrony and ostracized infant’s RMSSD during the experimental phase*

**Supplementary** **Table 1.** *Physiological reactivity (RMSSD) ANOVA*

| Measure | F | *p* | *η²p* |
| --- | --- | --- | --- |
|  |  |  |  |
| Phase (within) | 2.50 | .087 | .044 |
| Phase ✻ Condition | 8.57 | < .001* | .137 |
| Condition (between) | 0.792 | .377 | .014 |
| ^*^*p* < .05 |  |  |  |

**Supplementary** **Table 2.** *RMSSD Estimated Marginal Means*

|  |  |  |  |  | **95% CI** | |
| --- | --- | --- | --- | --- | --- | --- |
|  | **Condition** | **Phase** | **EMM** | **SE** | **LL** | **UL** |
| **RMSSD** | Inclusion | Free-play | 56.6 | 5.53 | 45.5 | 67.7 |
|  |  | Baseline | 62.2 | 5.65 | 50.9 | 73.5 |
|  |  | Experimental | 68.1 | 5.81 | 56.5 | 79.8 |
|  | Ostracism | Free-play | 59.3 | 5.53 | 48.2 | 70.4 |
|  |  | Baseline | 66.2 | 5.65 | 54.9 | 77.5 |
|  |  | Experimental | 43.7 | 5.81 | 32.0 | 55.3 |

**Supplementary** **Table 3.** *Behavioral Reactivity ANOVAs*

| **Positive Emotionality** | Measure |  | F | *p* | *η²p* |
| --- | --- | --- | --- | --- | --- |
|  |  |  |  |  |  |
|  | Phase (within) |  | 16.9 | <.001* | .228 |
|  | Phase ✻ Condition |  | 29.8 | <.001* | .344 |
|  | Condition (between) |  | 4.24 | .044* | .069 |
| **Negative Emotionality** | Phase (within) |  | 10.60 | .002* | .157 |
|  | Phase ✻ Condition |  | 5.94 | .018* | .094 |
|  | Condition (between) |  | 5.71 | .020* | .091 |
| **Active Engagement** | Phase (within) |  | 54.3 | <.001* | .488 |
|  | Phase ✻ Condition |  | 44.7 | <.001* | .440 |
|  | Condition (between) |  | 25.5 | <.001* | .309 |
| **Visual Attention** | Phase (within) |  | 2.4267 | .125 | .041 |
|  | Phase ✻ Condition |  | 0.0126 | .911 | .000 |
|  | Condition (between) |  | 0.0297 | .864 | .001 |
| **Look at the ball** | Phase (within) |  | 0.226 | .637 | .004 |
|  | Phase ✻ Condition |  | 8.192 | .006* | .126 |
|  | Condition (between) |  | 0.331 | .567 | .006 |
| **Look at the players** | Phase (within) |  | 5.26 | .026* | .084 |
|  | Phase ✻ Condition |  | 7.66 | .008* | .118 |
|  | Condition (between) |  | 0.493 | .485 | .009 |

**Supplementary** **Table 4.** *Behavioral reactivity Estimated Marginal Means*

|  |  |  |  |  | **95% CI** | |
| --- | --- | --- | --- | --- | --- | --- |
|  | **Condition** | **Phase** | **EMM** | **SE** | **LL** | **UL** |
| **Positive Emotionality** | Inclusion | Baseline | 23.9 | 2.57 | 18.77 | 29.1 |
|  |  | Experimental | 25.9 | 2.62 | 20.63 | 31.1 |
|  | Ostracism | Baseline | 24.8 | 2.62 | 19.57 | 30.0 |
|  |  | Experimental | 11.0 | 2.67 | 5.61 | 16.3 |
| **Negative Emotionality** | Inclusion | Baseline | 0.361 | 0.283 | -0.206 | 0.929 |
|  |  | Experimental | 0.893 | 1.024 | -1.158 | 2.943 |
|  | Ostracism | Baseline | 0.815 | 0.288 | 0.238 | 1.392 |
|  |  | Experimental | 4.513 | 1.042 | 2.427 | 6.599 |
| **Active Engagement** | Inclusion | Baseline | 3.02 | 0.609 | 1.80 | 4.24 |
|  |  | Experimental | 3.64 | 1.29 | 1.06 | 6.23 |
|  | Ostracism | Baseline | 2.52 | 0.620 | 1.28 | 3.76 |
|  |  | Experimental | 15.37 | 1.312 | 12.74 | 18.00 |
| **Look at the ball** | Inclusion | Baseline | 39.1 | 2.03 | 35.0 | 43.2 |
|  |  | Experimental | 35.2 | 2.40 | 30.4 | 40.0 |
|  | Ostracism | Baseline | 36.0 | 2.06 | 31.9 | 40.2 |
|  |  | Experimental | 41.5 | 2.44 | 36.6 | 46.3 |
| **Look at the players** | Inclusion | Baseline | 33.6 | 2.47 | 28.6 | 38.5 |
|  |  | Experimental | 34.3 | 2.46 | 29.4 | 39.3 |
|  | Ostracism | Baseline | 35.8 | 2.51 | 30.8 | 40.9 |
|  |  | Experimental | 27.7 | 2.50 | 22.7 | 32.7 |

**Supplementary** **Table 5.** *Linear regression between mind-attuned comments and infant’s RMSSD during the free-play phase*

| Effect | Estimate | *SE* | 95% CI | | *p* |
| --- | --- | --- | --- | --- | --- |
|  |  |  | *LL* | *UL* |  |
| (Intercept) | 67.504 | 5.538 | 56.38 | 78.627 | < .001* |
| Mind-attuned comments | -0.897 | 0.433 | -1.77 | -0.0268 | .044* |

**Supplementary** **Table 6.** *Linear regression between Sensitivity, Synchrony and ostracized infant’s RMSSD during the experimental phase*

| Effect | Estimate | *SE* | 95% CI | | *p* |
| --- | --- | --- | --- | --- | --- |
|  |  |  | *LL* | *UL* |  |
| (Intercept) | 43.3 | 18.14 | 5.742 | 80.79 | .026* |
| Sensitivity | 16.2 | 8.32 | -0.962 | 33.44 | .063 |
| Synchrony | -17.6 | 7.58 | -33.285 | -1.92 | .029* |
